# Intelligence, emotional intelligence, and emo-sensory intelligence: Which one is a better predictor of university students’ academic success?

**DOI:** 10.3389/fpsyg.2022.995988

**Published:** 2022-08-29

**Authors:** Reza Pishghadam, Maryam Faribi, Mahtab Kolahi Ahari, Farzaneh Shadloo, Mohammad Javad Gholami, Shaghayegh Shayesteh

**Affiliations:** Department of English, Ferdowsi University of Mashhad, Mashhad, Iran

**Keywords:** psychometric intelligence, emotional intelligence, emo-sensory intelligence, academic achievement, university students

## Abstract

The primary aim of this study was to determine the role of psychometric intelligence (IQ), emotional intelligence (EQ), and emo-sensory intelligence (ESQ) in university students’ academic achievement. To this end, 212 university students at different academic levels, composed of 154 females and 58 males, were asked to complete the Raven’s Progressive Matrices, the Bar-On Emotional Quotient Inventory, and the Emo-Sensory Intelligence Scale. Data were then matched with students’ Grade Point Averages as a measure of their academic achievement. The results revealed that students’ level of IQ and EQ could positively predict their academic achievement. In the case of their ESQ level, its auditory sub-component was found to be a positive predictor of academic success. Results were discussed, and possible implications and applications for increasing students’ chances for success were presented.

## Introduction

Achievement, in general, can be defined as how well individuals can perform quantitatively and qualitatively based on pre-set of facts and knowledge (Maramag-Manalastas and Batang, 2018; Lavrijsen et al., 2022). In the academic domain, this ability to perform is operationalized in terms of the outcomes that students achieve at the end of educational programs, which is called Academic Achievement (AA) and is associated with the active performance of academic skills (Ennser-Kananen et al., 2017; Peng and Kievit, 2020). More broadly speaking, AA can also refer to the acquisition of knowledge and skills through cognitive abilities (Mayer, 2011), which help students dwell well in educational tests and tasks; skills related to communication, literacy, science, thinking, social life, and mathematics (Lindholm-Leary and Borsato, 2006). Given the importance of AA in future success, there have been numerous attempts to find out which factors contribute to greater achievements in academic settings.

Earlier attempts focused mostly on cognitive factors, and since intelligence was recognized as a representation of cognitive abilities (Binet, 1905), it was considered the main predictor of AA (Kaya et al., 2015). Therefore, individuals with higher levels of IQ were considered educationally more successful (Pishghadam et al., 2020). Following the introduction of the emotional quotient (EQ) by Bar-On in 1997 and the sensory quotient (SQ) by Lombard (2007), the importance of emotional factors in AA gained momentum in research. Despite the lack of unanimity among early attempts, more recent views found that the level of EQ can strongly associate with and predict how much students can achieve in academic settings (Partido and Stafford, 2018; Denny et al., 2019; Suleman et al., 2019; MacCann et al., 2020; Sánchez-Álvarez et al., 2020). To further challenge the role of IQ in AA, researchers also found that SQ can outvalue IQ and EQ in predicting desirable performance in academia (Lombard, 2007), and more recently, ESQ (Pishghadam et al., 2020) has been recognized as a broader aspect of intelligence that integrates EQ with SQ. ESQ’s power was even more recognized when it was found as a significant predictor of English students’ Grade Point Average (GPA), with those possessing higher levels of ESQ maintaining higher GPAs (Pishghadam et al., 2020).

Cognition, emotions, and senses have all contributed to different theories of intelligence and consolidated their role in educational success. However, each theory has only proposed a fragmented view of intelligence and, thus, a one-sided view of factors affecting success in the educational domain. Academic achievement, however, is a multi-faceted combination of skills and abilities, and analyzing its association with each type of intelligence (IQ, EQ, or SQ) can unravel only one of its aspects. ESQ, on the other hand, can elaborate on AA more efficiently due to its broader perspective of intelligence; yet, more important than that is investigating how different types of intelligence associate with AA and predict it compared with one another. For university students, the significance of AA is undeniable since it plays a central role not only in university settings but in their prospective careers as well. Yet, it is still unclear how students can succeed academically. In other words, whether IQ, as an indicator of cognitive abilities, is essential in developing an academically-successful student or EQ and ESQ, as indicators of sensory/emotional abilities, is still a matter of debate. Drawing upon the triune theory of the brain (MacLean, 1990), we took the three major parts of the brain (i.e., rational, emotional, and sensory) into consideration and adopted a comparative approach to determine which type of intelligence among the three (i.e., IQ, EQ, and ESQ) plays a more significant role in academic gain and whether or not the combinatory nature of ESQ can be a more powerful predictor of AA. Therefore, this study postulates the following questions:

1. Is there any significant relationship between university students’ level of IQ, EQ, ESQ, and their AA?
2. Which type of intelligence can better predict university students’ AA?

## Review of literature

### Intelligence defined

Intelligence was first linked to cognitive abilities of logic and language, and the first psychometric test of intelligence (Binet, 1905) was designed to distinguish children with potential educationally-related mental deficits. Despite a lack of consensus among scholars in conceptualizing intelligence, most of them consider the following as the key components of intelligence: abstract thinking or reasoning, knowledge acquisition capacity, and problem-solving ability.

After the introduction of social intelligence by Thorndike, however, emotional and social aspects became integral components of intelligence. This recognition led to the introduction of EQ as a better predictor of success (Bar-On, 1988), encompassing skills to meet the demands of the surrounding social environment and rise above issues in life. To measure emotional intelligence, Bar-On (1997) developed an inventory of EQ, including both social and emotional competencies, called the Emotional Quotient Inventory (EQi). This inventory included five components: intrapersonal as emotional self-awareness and self-expression, interpersonal as awareness toward social relationships, adaptability as the ability to manage changes, stress management as emotional regulation competence, and general mood as an ability to keep oneself motivated (Bar-On, 1997). At the beginning of the 21st century, the importance of the body and senses in individuals’ cognition was once again recognized. Lombard (2007), therefore, extended the concept of intelligence to cover the additional ability of spotting, decoding, and monitoring sensory codes as sensory intelligence (SI) and SQ.

More recently, Pishghadam et al. (2020), drawing upon the concept of emotioncy (emotion + frequency; Pishghadam et al., 2013), adopted a combinatory approach to explaining more aspects of intelligence and proposed ESQ as a conciliation between EQ and SQ. The idea of emotioncy is defined as emotions created by sensory experiences which relativize cognition. Therefore, it combines sense, emotion, and cognition to shape a unified concept and build a bridge between felt experience and physical reality to clarify their relationship. Pishghadam et al. (2013; 2019; 2021; 2022) and Akbari and Pishghadam (2022) claimed that emotions are the byproducts of sensory experiences; therefore, what we perceive triggers emotional responses and creates reality. Accordingly, ESQ posits that intelligence is the ability to recognize, express, label, monitor, and manage sense-induced emotions; that is to say, cognition and perception are not solely constructed by the intellect; rather, it emerges from the blend of emotion and senses (Pishghadam et al., 2020). Based on this model, intelligence in separation (s factor) or in general (g factor) (Spearman, 1923) cannot cover all aspects of intelligence. EQ focuses on how well individuals can understand and manage their emotions regardless of what ignited these emotions. SQ describes individuals’ ability to connect with their senses and understand them, ignoring the mediation between senses and cognition. ESQ, on the other hand, draws a relationship between the two by considering emotions the mediator between senses and cognition, thereby emphasizing the role of senses in creating emotions, the centrality of emotions induced by senses, and that senses affect cognition through emotions they ignite. Individuals experience the world through their senses, which create in them certain emotions, and if they have a high level of ESQ, they can recognize these emotions and behave accordingly.

### Academic achievement and intelligence

Intelligence has always been the key cognitive factor explaining variations in AA (Kaya et al., 2015) and the subject of numerous studies examining how intelligence is related to educational success. The majority of these studies confirmed the association between general intelligence and AA (Jensen, 1998). However, the degree of this association has been inconsistent throughout research since different studies have found a range of moderate to strong correlations (0.40–0.63) existing between the two (Jencks, 1979; Macintosh, 1998). Very recently, IQ has been associated with more academic gains and found to be a powerful predictor of academic achievement (Guez et al., 2018; He et al., 2021). Therefore, IQ tests are still widely used to predict who can achieve more in academic contexts. More precisely, it was found that IQ can well predict how individuals perform in tests of reading, social sciences, natural sciences, and mathematics (Lohman, 2005; McCrocklin, 2020). However, evidence suggests that the verbal aspect of intelligence, which is related to the readiness to learn, has a stronger association with AA, compared to the non-verbal aspect of intelligence, which concerns the potential to learn (Kaya et al., 2015). Despite the rather unanimous findings of research regarding the role of IQ in AA, some recent attempts have shown contradictory findings. For example, in a study to investigate whether the academic performance of medical students is influenced by their IQ levels, Iqbal et al. (2021) found that since medical students are extremely hardworking, their level of IQ is not significantly associated with their AA in that no difference was noticed between the performances of more- and less-intelligent students. Therefore, the role of IQ in AA has recently been questioned.

To compensate for the lack of consistency in the relationship between IQ and AA, and following the recognition that IQ is not the sole predictor of academic success, researchers started to examine the association between EQ and AA. The majority of research in this regard has also found it a core competency that is positively correlated with and can predict individuals’ AA (Partido and Stafford, 2018; Denny et al., 2019; Suleman et al., 2019; MacCann et al., 2020; Pozo-Rico and Sandoval, 2020; Rajendran et al., 2020; Sánchez-Álvarez et al., 2020). Higher levels of EQ can improve students’ self-confidence and their ability to overcome challenges leading them toward better academic performance (Jan and Anwar, 2019). More precisely, EQ was found to act as a mediating variable between cognitive abilities and AA; therefore, a higher level of EQ was revealed to even facilitate the role that IQ plays in academic success (Petrides et al., 2004). Besides, it is worth mentioning that this association has been found to exist in all stages of life, such as primary education (Billings et al., 2014), secondary education (Sánchez-Álvarez et al., 2020), and primarily the university settings (Suleman et al., 2019). More recently, Pozo-Rico and Sandoval (2020) revealed that teachers who implemented EQ into their teaching plans could make a significant difference in their students’ AA, in that those with higher levels of EQ demonstrated higher GPAs compared with their less emotionally intelligent counterparts. They also found that the potential reason behind this difference in academic performance is that lower levels of EQ can be a hindrance to students’ motivation and can lead them to frequently procrastinate, which both negatively affect their results.

As suggested by Roy et al. (2013), since EQ is composed of different dimensions, each can, in turn, create competencies in students to make them more academically equipped. To further explain this association, studies began to investigate which specific dimensions of EQ are more strongly associated with and can better predict AA. In a longitudinal study, Parker et al. (2004) correlated first-year university students’ GPA with their EQ score to find which dimensions of EQ have a stronger role in AA. Their findings revealed that those with higher intrapersonal EQ, adaptability, and stress management abilities could cope with the demands of academic settings more successfully, thereby achieving higher GPAs. However, Fahim and Pishghadam (2007) focused only on English major students, and though confirming the association between intrapersonal, stress management, and AA, presented the third associative dimension as general mood competencies rather than adaptability. In another study, Fillipova and Bilyalov (2020) found that although self-management and intrapersonal components positively correlate with and predict AA, the interpersonal component poses a negative correlation, revealing that not all aspects of EQ can be a predictor of students’ success.

**FIGURE 1:**
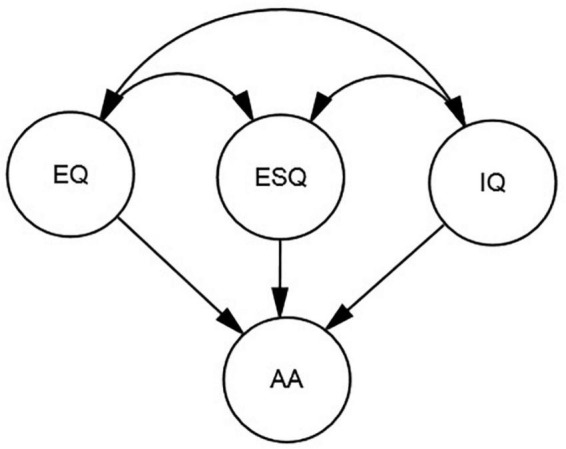
A hypothesized model for the possible relationships among IQ, EQ, ESQ, and AA.

Reviewing previous research clearly shows that there is a lack of consistent findings about how different types of intelligence can be associated with AA. More importantly, previous research has mostly focused on the two more widely-known aspects of intelligence (i.e., IQ and EQ), overlooking more recently-recognized types of intelligence such as ESQ. Therefore, the present study can fill this gap by clarifying which type of intelligence is more strongly associated with and can more powerfully predict university students’ level of success in academic settings. Figure 1 illustrates the possible relationships vividly.

## Methodology

### Participants

A total number of 212 university students, composed of 154 females and 58 males aging from 18 to 25 (M = 20.98, SD = 2.46) participated voluntarily in this study. The number of females exceeded that of the males owing to the fact that above 60% of university students in Iran are females. The participants were selected based on convenient sampling, and they all spoke Persian as their mother tongue. The participants were also asked to provide information related to their GPA from their university reports (M = 17.49, SD = 1.69) and degree (AA/S, *N* = 48); (BA/S, N = 129); (MA/S, *N* = 27); (Ph.D., *N* = 7).^1^ They provided written informed consent, and the study was approved by the Ferdowsi University of Mashhad Ethics Committee, Mashhad, Iran.

### Instrumentation

#### The Raven’s progressive matrices

Raven Matrices (Raven and Court, 1938) includes three versions, namely Standard Progressive Matrices (SPM), Colored Progressive Matrices (CPM), and Advanced Progressive Matrices (APM). The CPM is designed for children and less able adults, and APM targets 20% of the population (Raven, 2003). Thus, for the purpose of this study, the SPM version, comprising 60 items, which applies to the general population, was utilized. As a “well-validated measure of basic cognitive functioning” (Raven, 2000, p. 1), this test has been widely employed to determine an individual’s “capacity at the time of the test to apprehend meaningless figures presented for his observation, see the relations between them, conceive the nature of the figure completing each system of relations presented” (Raven et al., 1983, p. 2). The reason we used this test and not the more comprehensive ones measuring both fluid and crystalized intelligence was its feasibility to apply online during the COVID-19 pandemic.

#### The Persian version of Bar-On emotional quotient inventory

The Bar-On Emotional Quotient Inventory (EQ-i; Bar-On, 1997), as a self-report scale, aims to assess five areas of skills/competencies as follows: Intrapersonal, Interpersonal, Stress management, Adaptability, and General mood; and a 5-point Likert scale ranging from 1 (very seldom or not true of me) to 5 (very often or true of me) was used in this inventory for measuring participants’ EQ.

However, the Persian version of this inventory which includes 15 components and 90 items, was employed for the purpose of this study to ensure that our participants fully comprehended the questions since a possibility of misunderstanding was likely to be caused for some participants. In Samouei’s (2002) study, Cronbach’s alpha reliability for the translated version of this inventory was reported as 0.80.

#### The emo-sensory intelligence scale

The responsiveness to the emotions aroused by sensory inputs is considered the definition of Emo-Sensory Intelligence, which is of significance in modifying one’s behaviors and, as a result, can lead to success in life (Pishghadam et al., 2020). The emo-sensory intelligence scale, developed and validated by Pishghadam et al. (2020), is a 144-item scale for measuring emo-sensory intelligence consisting of 6 senses (auditory, visual, tactile, kinesthetic, smell, and taste) and 4 components (recognition, labeling, monitoring, and management). This scale (see Appendix 1 for sample items) uses a 5-point Likert scale ranging from 1 (very little) to 5 (very much) and was “validated through structural equation modeling, multitrait-multimethod design along with the Rasch measurement model” (Pishghadam et al., 2020, p. 173).

### Procedures

The three aforementioned tests were distributed online (using Google Forms) among the participants simultaneously. Each test took around 20–30 min for the participants to complete. The process of compiling the data lasted approximately 1 month (April to May 2021). A number of 240 forms were distributed among the participants, from which 220 were returned. Eight more forms were discarded due to invalid data. After the data were collected, the Pearson Product-Moment Correlation was run using the SPSS Software to determine the significance of the relationship among the intended variables. Then, the AMOS software was employed to run Structural Equation Modeling (SEM) and verify the relationship between the three types of intelligence (IQ, EQ, and ESQ) and AA.

## Results

This study sought to investigate whether there are any significant relationships between university students’ level of IQ, EQ, ESQ, and their AA, and which type of intelligence can better predict AA. The following sections represent the findings.

### Descriptive statistics

Descriptive statistics for AA and the IQ, EQ (intrapersonal, interpersonal, stress management, adaptability, and general mood), and ESQ (visual, olfactory, auditory, gustatory, tactile, and kinesthetic) measures can be seen in Table 1. Since the Skewness and Kurtosis estimates were within the range of −2 and +2, the normal distribution of the data was confirmed. Reliability coefficients were further calculated, which were all in an acceptable range.

**TABLE 1:** Descriptive statistics and reliability estimates for AA and the IQ, EQ, and ESQ scores.

	Min	Max	Mean	SD	Skewness	Kurtosis	Reliability
AA	10	20	17.49	1.69	–0.86	1.41	−
IQ	9	60	46.80	11.26	–1.51	1.69	0.78
EQ	181	428	326.71	45.46	–0.28	–0.08	0.95
Intrapersonal	1.83	4.77	3.60	0.54	–0.38	0.15	0.92
Interpersonal	2.22	5.00	4.10	0.54	–0.99	0.72	0.89
Stress management	1.50	4.67	3.08	0.74	0.12	–0.69	0.93
Adaptability	1.83	4.89	3.45	0.56	–0.03	0.00	0.88
General mood	1.25	5.00	3.80	0.70	–0.68	0.29	0.90
ESQ	232	680	499.59	73.14	–0.08	0.30	0.95
Visual	59	112	84.74	10.37	–0.011	–0.39	0.95
Olfactory	26	120	83.56	14.22	–0.30	0.76	0.89
Auditory	24	120	84.07	15.73	–0.13	0.91	0.92
Gustatory	38	120	84.02	14.37	0.13	–0.16	0.96
Tactile	29	120	82.96	15.43	–0.25	0.30	0.88
Kinesthetic	24	120	80.24	15.01	–0.40	1.83	0.91

### Correlational analysis

In order to find possible relationships between the variables of the study, Pearson Product-Moment Correlation was used. Based on Table 2, AA had a significant relationship with IQ (*r* = 0.18, *p* < 0.01), the overall EQ (*r* = 0.12, *p* < 0.05), and its general mood subconstruct (*r* = 0.12, *p* < 0.05), and the auditory subconstruct of ESQ (*r* = 0.14, *p* < 0.05). EQ and four of its subconstructs, namely intrapersonal, interpersonal, adaptability, and general mood, were positively correlated with ESQ and all of its subconstructs. Stress management, however, was positively correlated with ESQ (*r* = 0.13, *p* < 0.05) and two of its subconstructs namely visual (*r* = 0.15, *p* < 0.05) and kinesthetic (*r* = 0.16, *p* < 0.05).

**TABLE 2:** Correlational analysis for the variables.

	1	2	3	4	5	6	7	8	9	10	11	12	13	14	15
AA	1														
IQ	0.18**	1													
EQ	0.12*	0.06	1												
Intra	–0.07	0.08	0.92**	1											
Inter	–0.00	0.06	0.74**	0.58**	1										
Stress	–0.05	0.02	0.76**	0.62**	0.43**	1									
Adapt	–0.03	0.08	0.86**	0.78**	0.49**	0.62**	1								
Mood	0.12*	–0.04	0.87**	0.77**	0.67**	0.56**	0.66**	1							
ESQ	0.06	0.08	0.31**	0.34**	0.23**	0.13*	0.32**	0.23**	1						
Visual	0.08	0.00	0.26**	0.25**	0.27**	0.15*	0.24**	0.15*	0.69**	1					
Olfactory	0.03	0.06	0.31**	0.35**	0.24**	0.13	0.32**	0.21**	0.88**	0.65**	1				
Auditory	0.14*	0.04	0.26**	0.30**	0.14*	0.11	0.31**	0.18**	0.88**	0.51**	0.79**	1			
Gustatory	0.06	0.11	0.23**	0.25**	0.16*	0.09	0.26**	0.15*	0.91**	0.51**	0.74**	0.81**	1		
Tactile	0.02	0.10	0.23**	0.26**	0.17*	0.06	0.22**	0.20**	0.88**	0.51**	0.69**	0.70**	0.79**	0.1	
Kinesthetic	0.01	0.06	0.30**	0.31**	0.22**	0.16*	0.28**	0.26**	0.84**	0.49**	0.63**	0.64**	0.75**	0.76**	1

**Correlation is significant at the 0.01 level (2-tailed). *Correlation is significant at the 0.05 level (2-tailed). AA, Academic Achievement; Intra, Intrapersonal; Inter, Interpersonal; Stress, Stress Management; Adapt, Adaptability; Mood, General Mood.

### Structural equation modeling analysis

**FIGURE 2:**
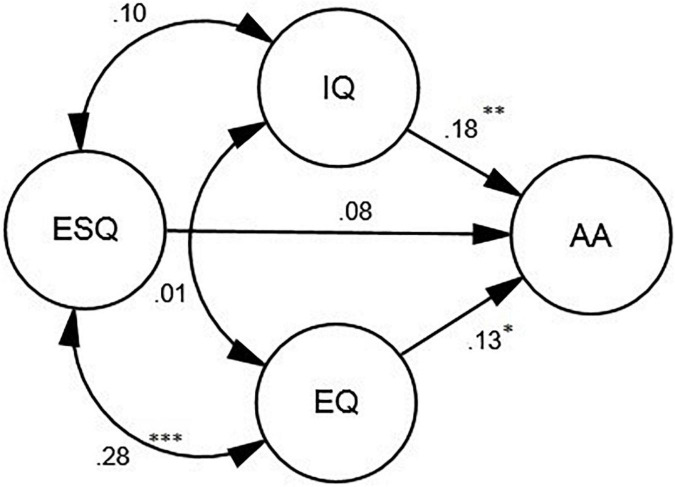
The schematic representation of the relationships between the three types of intelligence (i.e., IQ, EQ, and ESQ) and Academic Achievement (AA). *p < 0.05, ^**^p < 0.01, ^***^p < 0.001.

A SEM model was conducted to verify the relationship between the three types of intelligence and AA. The goodness of fit indices showed that the model fits the data adequately (see Table 3). According to Figure 2, IQ (β = 0.18, *p* < 0.01, *R*^2^ = 0.05) and EQ (β = 0.13, *p* < 0.05, *R*^2^ = 0.05) were the positive predictors of AA. Yet, ESQ could not significantly predict AA.

**TABLE 3:** Goodness of fit indices for the models.

Model	χ ^2^/df	df	CFI	TLI	IFI	GFI	RMSEA	SRMR
Figure 2	1.82	56	0.97	0.96	0.97	0.93	0.06	0.05
Figure 3	2.53	1	0.96	0.95	95	0.92	0.05	0.03
Figure 4	1.24	1	0.96	0.96	0.95	0.90	0.05	0.03

**FIGURE 3:**
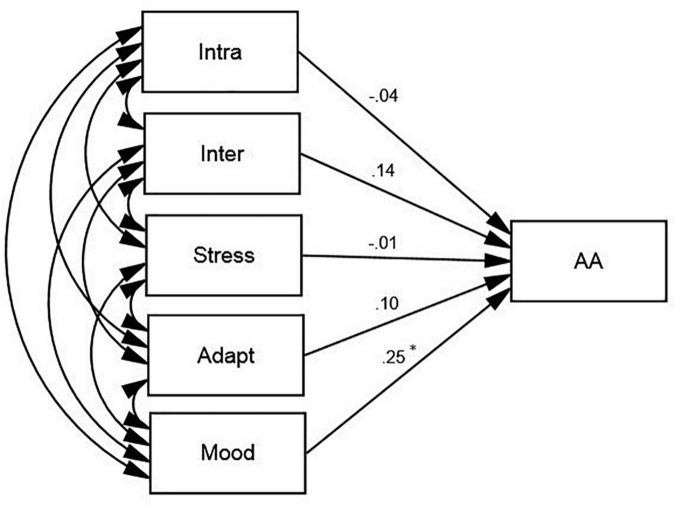
The schematic representation of the relationships between Academic Achievement (AA) and the subconstructs of EQ (Intra, Intrapersonal; Inter, Interpersonal; Stress, Stress Management; Adapt, Adaptability; Mood, General Mood; *p < 0.05).

**FIGURE 4:**
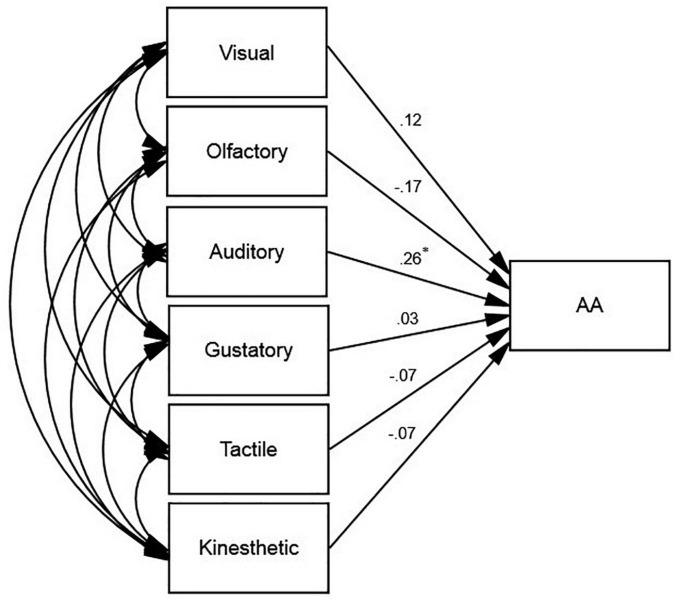
The schematic representation of the relationships between Academic Achievement (AA) and the subconstructs of ESQ, namely visual, olfactory, auditory, gustatory, tactile, and kinesthetic (*p < 0.05).

In order to check the predictive power of the subconstructs of EQ and ESQ, two more models were proposed (Figures 3, 4), which fitted the data adequately (see Table 3). As Figure 3 illustrates, among the subconstructs of EQ, general mood was a positive predictor of AA (β = 0.25, *p* < 0.05, *R*^2^ = 0.03).

According to Figure 3, among the subconstructs of ESQ, auditory (β = 0.26, *p* < 0.05, *R*^2^ = 0.03) was the only predictor of AA.

## Discussion

The main aim of this study was to investigate the association between the three types of intelligence (i.e., IQ, EQ, and ESQ) and university students’ AA. Additionally, this study tried to determine which type of intelligence has stronger power to predict AA, whether it is IQ, as a representation of cognitive ability, EQ, as a representation of social-emotional competence, or ESQ, as an interplay between EQ and SQ.

In response to the first research question, we aimed to look into the relationship between IQ and AA. Participants’ answers to the first questionnaire revealed that IQ has a strong and positive relationship with AA. The findings also revealed that IQ could strongly predict academic success and achievement. Therefore, individuals with higher levels of IQ were more academically successful, and their level of academic success could be predicted based on their IQ level. Previous attempts found moderate (Gottfredson, 2005) to strong (Jensen, 1998; Lohman, 2005; Guez et al., 2018; He et al., 2021) correlations between the two variables, but the findings of the present study confirmed only a weak yet significant correlation. The low correlation suggests that over the past decades, IQ seems to have lost its strong association with AA due to environmental factors, social status, influences of gender, and even the COVID-19 pandemic. Yet, more research needs to be done to confirm this claim.

Regarding the relationship between EQ and AA, participants’ responses to the Bar-On test of EQ revealed that students’ overall level of EQ and its general mood sub-component were strongly and positively correlated with AA and had a significant predictive validity. That is, students tend to perform better in university settings when they can functionally understand and regulate their emotions. The finding could confirm previous attempts about the association between the overall EQ score and AA (Partido and Stafford, 2018; Denny et al., 2019; Suleman et al., 2019; MacCann et al., 2020; Pozo-Rico and Sandoval, 2020; Rajendran et al., 2020; Sánchez-Álvarez et al., 2020); however, regarding the sub-components, while other studies found positive associations between intrapersonal, adaptability, stress management, and general mood sub-components and English language students’ AA (Parker et al., 2004; Fahim and Pishghadam, 2007), the findings of this study could only confirm the association between general mood and AA. It can imply that students are more academically successful only if they are in a good mood which might be generated in classroom settings. A possible reason for the dissimilarity of the findings could be the different sample populations and the nature of the students’ majors.

Except for its auditory subconstruct, no significant correlation was found between ESQ and AA. In fact, ESQ seems to be a young theory in need of more theoretical and empirical research and training to clarify its association with AA or other educational domains. The representation of the relationships of ESQ and its sub-components with AA revealed that the auditory sub-component was positively and strongly related to and could predict AA. That is, university students who were high academic achievers were more auditory-based. Inevitably, the sense of hearing seems to be more active in these students since the educational system in Iran focuses more on auditory teaching styles and lectures, and thus, most of what they need to learn comes from auditory sources. As a result, the more active their auditory sense, the better they can learn and perform in academic settings. This finding was partially in line with that of Pishghadam et al. (2020), who found associations between visual and kinesthetic sub-components and English language students’ GPA. A possible reason for the inconsistency in their finding could be the major of the participants. While the participants of Pishghadam et al.’s (2020) study were English language university students, the participants of this study had miscellaneous majors.

Considering the second research question, it was found that the students’ level of IQ is more strongly associated and can best predict their academic achievement compared with other intended intelligence types in this study. It can be concluded that in traditional educational systems like that of Iran, the main focus is still on the stereotypical understanding of intelligence which is the representation of cognitive ability or IQ. This trend represents itself in teaching and testing practices which mutually assume that students with higher IQ levels stand better chances of success. More importantly, it is implied that other types of intelligence, such as EQ, are still new in the context of Iran and need to be more focused upon by the educational system so that this ability also gets developed and contributes to academic success even more. Finally, the lack of association between ESQ and AA in this study reveals that senses and their induced emotions are not well-recognized in the context of Iran, and the educational system has not yet invested in developing this capability of individuals.

### Implications

The results of this study can be practical for different groups of individuals. First, teachers are expected to be more familiar with the concepts of IQ and EQ. Moreover, curricula should seek to educate learners about the value of IQ, EQ, and ESQ. Material developers are required to include practices with at least a peripheral focus on EQ and ESQ, which can enable learners to discover other aspects of their intelligence. It also seems necessary to pay attention to senses other than auditory by including other sub-component of ESQ in our academic context to promote them in learners and suit a wider range of learning styles. Finally, test developers can also benefit from the findings of this study such that in testing individuals’ academic success, they can also tap into aspects of intelligence such as EQ and ESQ. In this way, students are indirectly informed that success in tests is not limited to IQ only.

### Limitations and suggestions for future research

This study can be improved if the following issues are taken into account. One limitation is that this study focused on fluid intelligence only. Future research can apply more comprehensive models of IQ, such as Cattell–Horn–Carroll (CHC), given that intelligence is a relative concept with multiple aspects and cannot be covered by a single theory. Moreover, this study did not take age, gender, and major into account, thereby, generalizing the findings of this study across different ages, genders, or majors is subject to certain limitations. Further studies regarding the role of SQ would be useful as well. Finally, similar studies to this one can be conducted in other settings to compare the results and determine the extent to which the associations between IQ, EQ, ESQ, and AA differ in different settings.

## Data availability statement

The raw data supporting the conclusions of this article will be made available by the authors, without undue reservation.

## Ethics statement

This study was reviewed and approved by the Ferdowsi University of Mashhad Ethics Committee, Mashhad, Iran. The participants provided written informed consent to participate in this study.

## Author contributions

RP conceived and designed the experiments. MF, MK, FS, and MG performed the experiments. SS and RP analyzed the data, contributed reagents, materials, and analysis tools, and reviewed and edited the manuscript. MF, MK, FS, MG, and SS wrote the manuscript. All authors contributed to the article and approved the submitted version.

## Appendix

Appendix 1 ∣ Sample items from the emo-sensory intelligence scale.

1. I know the sounds that make me feel sad (Auditory, Recognition).
2. Expressing my feelings toward images that are surprising is hard for me (Visual, labeling).
3. I can control and monitor the sorts of smells that have disgusted me in the past (Olfactory, Monitoring).
4. Refraining from touching things that frighten me is hard for me (Tactile, Management).

^1^AA, Associate of Arts; BA, Bachelor of Arts; MA, Master of Arts; Ph.D., Doctor of Philosophy.
